# The Roles of Non-coding RNA in the Development and Regeneration of Hair Follicles: Current Status and Further Perspectives

**DOI:** 10.3389/fcell.2021.720879

**Published:** 2021-10-11

**Authors:** Min Yang, Tingting Weng, Wei Zhang, Manjia Zhang, Xiaojie He, Chunmao Han, Xingang Wang

**Affiliations:** ^1^Department of Burns & Wound Care Center, Second Affiliated Hospital of Zhejiang University, Hangzhou, China; ^2^Key Laboratory of the Diagnosis and Treatment of Severe Trauma and Burn of Zhejiang Province, Hangzhou, China; ^3^The First Clinical Medical College, Zhejiang Chinese Medical University, Hangzhou, China; ^4^Department of General Practice, Second Affiliated Hospital of Zhejiang University, Hangzhou, China

**Keywords:** hair follicle, hair follicle cycling, non-coding RNA, regeneration, alopecia

## Abstract

Alopecia is a common problem that affects almost every age group and is considered to be an issue for cosmetic or psychiatric reasons. The loss of hair follicles (HFs) and hair caused by alopecia impairs self-esteem, thermoregulation, tactile sensation and protection from ultraviolet light. One strategy to solve this problem is HF regeneration. Many signalling pathways and molecules participate in the morphology and regeneration of HF, such as Wnt/β-catenin, Sonic hedgehog, bone morphogenetic protein and Notch. Non-coding RNAs (ncRNAs), especially microRNAs and long ncRNAs, have significant modulatory roles in HF development and regeneration via regulation of these signalling pathways. This review provides a comprehensive overview of the status and future prospects of ncRNAs in HF regeneration and could prompt novel ncRNA-based therapeutic strategies.

## Introduction

Hair follicles (HFs), which protrude from mammalian skin and are considered to be mini-organs, are formed via epidermis–dermis interactions in the embryo (Schmidt-Ullrich and Paus, 2005). Due to the presence of stem cells, HF periodically regenerate to produce keratinised hair continuously throughout life (Rompolas and Greco, 2014). Hairs grow from HFs and absorb nutrients from the body to support their growth. Decreases in HF regeneration are a result of aging and some diseases, cause alopecia (Schneider et al., 2009). Hair is not only important for aesthetic reasons, but also plays a crucial role in thermoregulation, tactile sensation and protection from ultraviolet (UV) light (Millar, 2015; Brown and Krishnamurthy, 2020).

Alopecia is a common problem that affects almost every age group. It is considered to be an issue for cosmetic or psychiatric reasons, especially in women. Alopecia is typically categorised as scarring alopecia or non-scarring alopecia, and the latter can be further divided into seven subtypes according to the systemic cause, such as alopecia areata or androgenetic alopecia (Phillips et al., 2017). Among Caucasians, the morbidity rate for androgenetic alopecia is roughly 45% (Norwood, 1975; Gan and Sinclair, 2005), while in China, the rates are 21.3% and 6% for males and females, respectively (Xu et al., 2009; Wang et al., 2010). This indicates that hair loss is a serious problem that must be solved urgently. The diagnosis and clinical treatment of alopecia must be improved, because most therapeutic strategies available today are palliative.

Non-coding RNAs (ncRNAs) are ubiquitous throughout the human genome. Unlike mRNAs, ncRNAs do not encode proteins, although they do play a significant modulatory role in various biological processes, such as cell proliferation, the cell cycle, epigenetic modification and apoptosis (Yang et al., 2019). There are several types of ncRNA, including small nuclear RNAs, small nucleolar RNAs, ribosomal RNAs, large intergenic ncRNAs, microRNAs (miRNAs) and long ncRNAs (lncRNAs) (Liu et al., 2020). The ncRNAs modulate gene expression by serving as transcriptional and post-transcriptional regulators within complex regulatory networks (Anastasiadou et al., 2018; Panni et al., 2020). Accumulating evidence indicates that several ncRNAs are associated with the development and regeneration of HFs (Lin et al., 2014; Jiao et al., 2019; Ma et al., 2019). Increasing our understanding of the roles of ncRNAs in HFs could inspire novel strategies for developing ncRNA-based therapeutics.

## The Anatomy, Morphogenesis, and Cycling of Hair Follicles

Hair follicles are dynamic mini-organs and their development has been studied extensively (Schneider et al., 2009). HFs and keratinised hair are present on most of the body’s surface, except for the palms, plantar, lips, papilla and parts of the urogenital tissues. The distribution, quantity and texture of hair are primarily driven by sex hormones.

### Hair Follicle Anatomy

**FIGURE 1:**
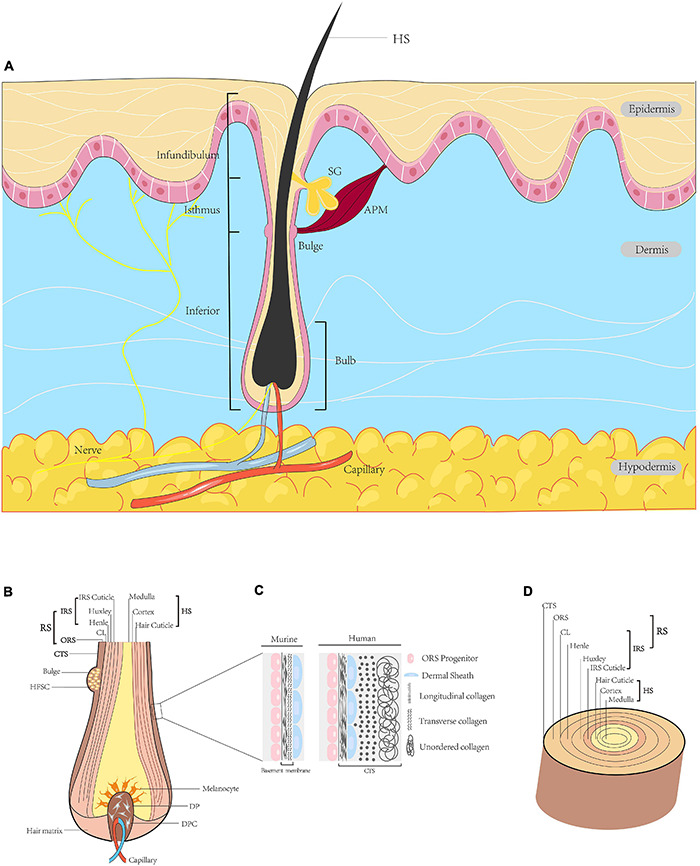
Diagram of HF. **(A)** Schematic illustration of full-thickness skin in sagittal section. HFs are located in the dermis, with the hair shaft (HS) extruding above the epidermis. The HF is divided into three segments, i.e., the infundibulum, isthmus and inferior segment. The intumescent part at the bottom of the HF is the bulb. At the insertion site of the arrector pili muscle, a convex protrusion of the outer root sheath (ORS) forms a bulge containing many HFSCs. The duct of the sebaceous gland opens directly into the HF. **(B)** Sagittal section of the bulb. The centre of the bulb is the DP surrounded by hair matrix, consisting of specialised mesenchymal fibroblasts (DPCs) and extracellular matrix. The bulb contains melanocytes, hair matrix cells and ORS cells. **(C)** Higher magnification of the region indicated. In the human scalp, this layer is the connective tissue sheath (CTS), in which the dermal sheath (DS) is between longitudinal and transverse collagen. In the murine pelage, this layer forms the basement membrane, with the DS on the outside. **(D)** Transverse section of the HF. From outside to inside: CTS (in human), RS and HS. The RS is subdivided into the ORS and IRS. The IRS is composed of the CL, Henle’s layer, Huxley’s layer and IRS cuticle. The outermost layer of the HS is the hair cuticle, followed in order by the cortex and the medulla (Paus and Cotsarelis, 1999; Al-Nuaimi et al., 2010; Wang et al., 2012; Martino et al., 2021). HF, hair follicle; HS, hair shaft; APM, arrector pili muscle; ORS, outer root sheath; HFSC, hair follicle stem cell; SG, sebaceous gland; DP, dermal papilla; DPC, dermal papilla cell; CTS, connective tissue sheath; DS, dermal sheath; RS, root sheath; IRS, inner root sheath; CL, companion layer.

Hair follicles arise from reciprocal interactions among ectoderm-mesoderm tissues, and are comprised of eight cell layers (Figures 1B,D). HFs contain multiple cell types, such as melanocytes and dermal papilla cells derived from the neural crest, ectoderm or mesoderm. These cells vary in location, function and gene expression profile (Al-Nuaimi et al., 2010). HFs are situated in the dermis of the skin or within invaginations of the epidermis. Mature HFs consist of a mesenchymal part, including dermal papilla (DP) and a perifollicular connective tissue sheath (CTS), and an epithelial part containing the hair matrix, hair shaft and root sheath (RS). HFs can be divided into three segments: the infundibulum (top layer) from the sebaceous gland opening to the epidermis surface; the isthmus (middle layer) from the sebaceous gland to the bulge at the insertion of the arrector pili muscle; and the inferior segment (bottom layer). The arrector pili muscle connects the HF and adjacent dermis (Wang et al., 2012); cold stimuli induce the arrector pili muscle to contract, which orients hair vertically. The DP is surrounded by the hair matrix at the base of the HF, which contains an abundant neurovascular network that provides nourishment to the HF and supports its sensory function (Weng et al., 2020). Matrix and progenitor cells proliferate and spread throughout the bulb and dermal sheath (DS) (Dong et al., 2012). Matrix cells act as germ cells and participate in HF cycling, while progenitor cells play a role in papilla regeneration and wound healing. The RS, which begins at the epidermis, can be divided into two parts, i.e., the outer root sheath (ORS) and the inner root sheath (IRS). The bulge is located in the ORS, where the arrector pili muscle is inserted. The IRS can be further subdivided from inside to outside into the cuticle, Huxley layer, Henle layer and companion layer (CL). The cuticle is composed of squamous cells in direct contact with the hair shaft, thus contributing to the close relationships among the IRS, hair shaft and keratin production (Hardy, 1992; Brown and Krishnamurthy, 2020; Figure 1).

### Hair Follicle Morphogenesis

**FIGURE 2:**
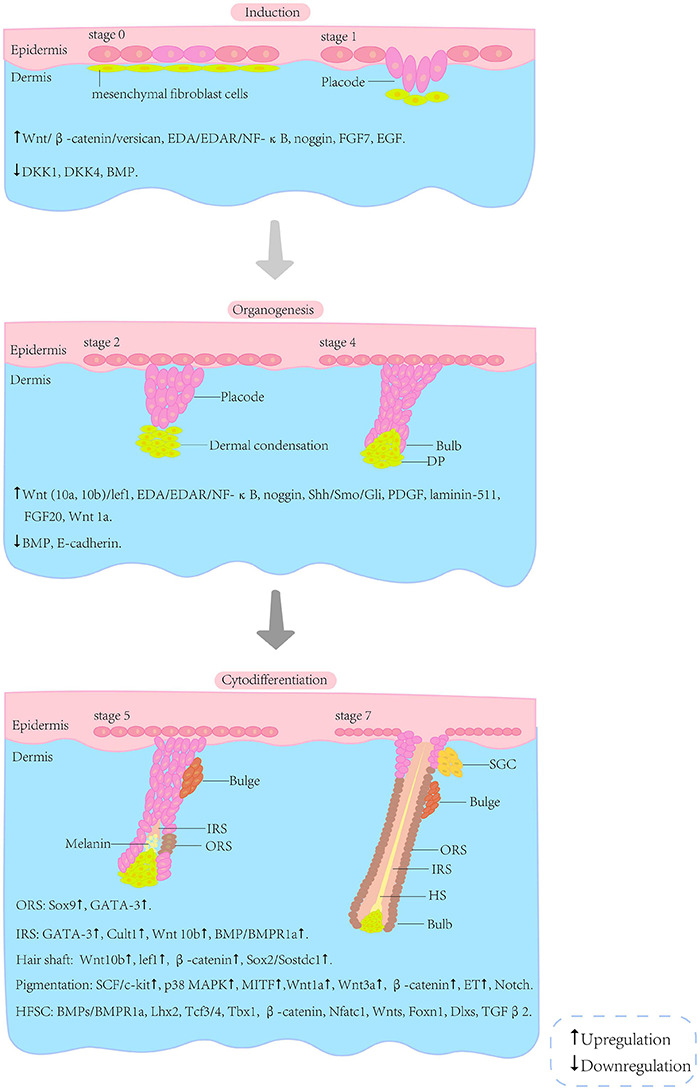
Schematic illustration of selected stages of HF morphogenesis. The morphogenesis of the HF can be roughly divided into three phases (i.e., induction, organogenesis and cytodifferentiation) and eight stages. The boundaries between these phases are not clear. The interactions associated with the phases are listed below the illustration (Paus and Cotsarelis, 1999; van der Veen et al., 1999; Schneider et al., 2009; Shimomura and Christiano, 2010; Rishikaysh et al., 2014). HF, hair follicle; HFSC, hair follicle stem cells; SGC, sebaceous gland cells; DP, dermal papilla; IRS, inner root sheath; ORS, outer root sheath; HS, hair shaft.

The embryonic development of HF arises from the reciprocal regulation of mesenchymal and epithelial cells; this process involves many signalling pathways, such as Wnt/β-catenin, Sonic hedgehog (Shh), bone morphogenetic protein (BMP) and Notch (Rishikaysh et al., 2014). Although many signals and molecules involved in HF morphogenesis have been identified, further investigations are required to gain a complete understanding of the complex network. The morphogenesis of HF can be roughly divided into three phases, i.e., induction, organogenesis and cytodifferentiation (Figure 2). However, the boundaries between these phases are not clear.

#### Hair Follicle Induction

The initial signal for HF induction comes from the dermis and is received by epithelial cells (Sennett and Rendl, 2012). Experiments performed using the Wnt/β-catenin inhibitor Dkk 1 revealed the essential role of Wnt/β-catenin in the initiation of HF induction and placode formation (Andl et al., 2002).

First, specialised mesenchymal fibroblasts gather below the epidermis. This aggregative phenomenon is directly associated with regulation of versican expression by Wnt/β-catenin (Yang et al., 2012). Melanocytes derived from the neural crest begin migrating upward to the epidermis (Qiu et al., 2019). The first specific Wnt triggering the initiation is still obscure, although Wnt5a is known to be the second one. Then, stable activity of β-catenin in the upper dermis directs thickening of the epithelium to form the placode, and the signals in turn affect the fibroblasts beneath the epidermis (Hardy, 1992). There exists not only an interaction between fibroblasts and placode, but also crosstalk of the placode with its surroundings.

Ectodysplasin-A (EDA)/EDA receptor (EDAR)/nuclear factor-kappa B (NF-κB) is another essential signalling pathway for primary placode maintenance (Srivastava et al., 2001). Although the initial role of Wnt is independent of NF-κB activation, abnormal placode with an irregular border and fused or string-like structure occurs when NF-κB is absent. Sick et al. (2006) reported that Dkk4 exerted its regulatory effects on the size and space of the placode by combining with NF-κB. NF-κB is regulated by Wnt/β-catenin signalling, and in turn modulates Wnt10b directly to refine the border of the placode. This discovery suggested reciprocal effects among Dkk4, Wnt and NF-κB in the initiation and maintenance of the primary placode (Laurikkala et al., 2002; Sick et al., 2006; Zhang et al., 2009). NF-κB also acts as a suppressor of BMP, which is part of the transforming growth factor-β (TGF-β) signalling superfamily (Dinter et al., 2019). Mice overexpressing the BMP antagonist noggin (k14-noggin mice) exhibit higher HF densities than wild-type controls. In addition, in EDA-null mice, β-catenin overexpression triggers primary placode formation. However, chronic β-catenin activation causes abnormal HF formation, which may be a result of interference with BMP signalling (Narhi et al., 2008). Noggin can partially rescue placode formation, suggesting that BMP suppresses HF development and that placode formation is a result of the balance between activated and inhibitory signals (Plikus et al., 2004). In addition to these signalling pathways, other molecules such as fibroblast growth factor 7 (FGF7 or KGF) and epidermal growth factor (EGF) are also required for the initiation of HF. The receptors of both factors are downregulated while the endogenous ligands are expressed throughout the initiation period (Richardson et al., 2009).

#### Hair Follicle Organogenesis

Placode formation is followed by dermal condensation (DC) formation and placode growth. The formation of DC, the precursor of DP, is the key step in HF organogenesis (Rishikaysh et al., 2014). Its morphogenesis is dependent on the regulation of directed fibroblast migration by fibroblast growth factor (FGF) 20 (Biggs et al., 2018). Wnt/β-catenin induces fibroblasts to assemble DC and regulates the size of DC by activating dermal progenitors (Gupta et al., 2019). EDA/EDAR/NF-κB signalling plays a pivotal role in HF organogenesis. It functions through the Shh signalling pathway to promote cyclin D1 expression (Sima et al., 2018). Shh-knockout mice are completely hairless, although primary and secondary HF germs are formed. In addition, Shh is unable to fully compensate for the lack of EDA in Shh-overexpressing Tabby mice (Cui et al., 2011). These results verified the EDA-Shh cascade. In Shh-null HF, epithelial proliferation is inhibited, while its differentiation is unaffected. The target genes of Shh are expressed in both the epithelial and mesenchymal compartments (DC), suggesting that both compartments receive Shh signals. Sun et al. (2020) showed that Shh in adjacent epithelial cells and stromal cells could induce *de novo* HF regeneration in hairless paw skin, and that single Shh signalling in specific cell types was sufficient to reactivate HF regeneration in unwounded adult tissue. Shh is not necessary for HF initiation, but is essential for epithelial proliferation and HF downgrowth. Both noggin and Shh are secreted from placode. Shh is crucial for the maturation of DP and its sustained expression depends on BMP inhibition, which is mediated via noggin (Dahmane et al., 1997; Huntzicker et al., 2006; Woo et al., 2012). This represents a complex interaction between epithelial and mesenchymal compartments involving many molecules, including epithelial platelet-derived growth factor (PDGF) and epithelial laminin-511. Noggin expression relies on Shh, and in turn influences Shh via inhibition of BMP by Lef1 expression. Epithelial laminin-511 interacts with β integrin to activate the downstream targets (smoothened, Gli, etc.) of Shh (Rishikaysh et al., 2014). The interplay between noggin and BMP results in downregulation of E-cadherin in the placode. Overexpression of E-cadherin in the epidermis leads to HF deficiency in transgenic mice, suggesting that E-cadherin is crucial for placode downgrowth (Jamora et al., 2003).

In the later period of organogenesis, DC further develops into DP. Subsequently, cells from the epithelial compartment differentiate into specific tissues, such as IRS, ORS, etc. Wnt1a maintains the characteristics of DP cells (DPCs) and promotes HF regeneration (Dong et al., 2014).

#### Cytodifferentiation

The most obvious characteristic of this phase is the differentiation of cells among epithelial and mesenchymal compartments, which involves numerous signalling pathways and molecules. Shh takes part in the differentiation of RS via activation of its downstream target, smoothened (Gritli-Linde et al., 2007). Sox9 is a downstream target of Shh, first detected in placode during HF morphogenesis, and then expressed in ORS and bulge. In the absence of Sox9, hair loses the ability to proliferate and the HF fails to develop the niche of stem cells (Vidal et al., 2005). GATA-3 is expressed in the initial stages of epidermal stratification and IRS differentiation. IRS progenitors fail to differentiate, and abnormal hair is produced in the GATA-3-null HF, suggesting a crucial role of GATA-3 in the IRS (Kaufman et al., 2003). Cutl1 is essential for epithelial differentiation of the HF, and Cutl1 mutant mice show reduced IRS and aberrant pelage (Ellis et al., 2001). Moreover, Wnt10b promotes the differentiation of primary skin epithelial cells into IRS, causing elongation of the hair shaft (Ouji et al., 2007). The regulation of IRS mentioned above can be summarised as follows: BMPs activate Bmpr1a (the only known BMP receptor expressed in the HF), upregulating GATA-3 and modulating IRS progenitor differentiation or maintaining sufficient lef1 and stabilised β-catenin to regulate hair shaft growth. Dlx3 can also control the differentiation of IRS and hair shaft as a target of lef1 (Kobielak et al., 2003; Nemer and Nemer, 2003; Hwang et al., 2008). Hair shaft growth is also regulated by upregulation of BMP6 and downregulation of Sostdc1 by Sox2 (Clavel et al., 2012). Notch exerts its effects on cell fate, stem cell potentiality and differentiation, cell adhesion regulation, epidermal cell localisation and cellular differentiation promotion by suppressing p63 (Nguyen et al., 2006; Watt et al., 2008). Notch 1 activates Wnt5a expression in DP, thus promoting FoxN1 expression, which plays a significant role in regulating keratinocyte differentiation and inducing melanin secretion from melanocytes to keratinocytes localised in the hair cortex (Weiner et al., 2007).

### Hair Follicle Cycling

**FIGURE 3:**
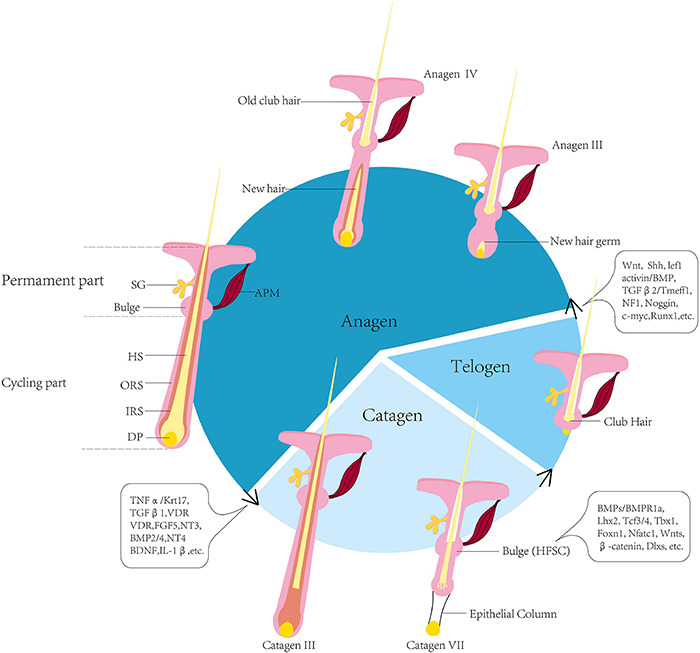
Diagram of selected stages of the HF cycle. The HF cycle can be divided into three phases, i.e., anagen, catagen, telogen. Once it matures, the HF enters into catagen to begin the cycle, following the activation of numerous signals. In catagen, the lower 2/3 of the HF regresses due to apoptosis in the IRS, ORS and hair matrix, and eventually forms the epithelial column. The DP moves up and is in close proximity to the bulge. In the telogen, the primary HS transforms into the club hair, and proximity between the DP and bulge is maintained. The interaction between the bulge and DP activates the transition to anagen. The stem cells in the upper 1/3 of the HF are activated. The HF grows downward to construct the bulb and other structures. New hair is generated, and the old club hair is shed. The signalling pathways involved in the transition are indicated in the bubbles (Paus and Cotsarelis, 1999; Al-Nuaimi et al., 2010; Shimomura and Christiano, 2010; Houschyar et al., 2020). HF, hair follicle; IRS, inner root sheath; ORS, outer root sheath; DP, dermal papilla; HS, hair shaft; HFSC, hair follicle stem cell; SG, sebaceous gland; APM, arrector pili muscle.

Hair follicle cycling is the process by which mature HFs regenerate. The morphology and gene expression profiles of HF undergo periodic changes in three phases, i.e., anagen, catagen and telogen phases. Only the lower ∼2/3 of the HF undergoes this cycling, while the upper 1/3 maintains its structure (Paus and Cotsarelis, 1999; Paus and Foitzik, 2004). In the anagen phase, or growth phase, the upper part grows downward and the matrix region is formed. The matrix cells from this region proliferate, differentiate and migrate to form the IRS and hair shaft (Langbein and Schweizer, 2005). Melanin is produced by melanocytes in the bulb, and hair pigmentation occurs during this phase. Insulin-like growth factor 1 (IGF-1) and fibroblast growth factor-7 (FGF-7), which are secreted by DPs during the anagen phase, play key roles in anagen maintenance (Paus and Cotsarelis, 1999). In the catagen phase, the hair matrix, IRS and ORS undergo apoptosis, and the lower part of the HF rapidly degrades toward the upper part. Apoptosis is prevented in the DP due to the synthesis of the anti-apoptosis protein, B-cell lymphoma-2 (Bcl-2) (Botchkareva et al., 2006). DPCs transform into a cluster of quiescent cells and the DP eventually remains in close proximity to the bulge. During the telogen stage, the activities of cells (e.g., DPCs, HFSCs, epithelial cells) in the HF are greatly attenuated. The club hair derived from the hair shaft is eventually shed (Schneider et al., 2009). Various regulatory factors and metabolites, such as α-ketoglutarate and α-ketobutyrate, are associated with the telogen phase and the activation of autophagy (Chai et al., 2019; Figure 3).

Following the interplay among DPCs and HFSCs, involving Wnt, BMP and TGF, the next cycle begins and repeats (Alonso and Fuchs, 2006). At the beginning of telogen, BMP imposes a threshold of activation that must be surmounted to induce the transition from the telogen to anagen phase. The TGF-β2 signalling pathway is essential in HF cycling for activation of smad2/3 in HFSCs, to avoid delayed regeneration. The TGF-β2 pathway promotes telogen–anagen transition by activating Tmeff1, which can lower the threshold of activation (Oshimori and Fuchs, 2012). Shh also facilitates this transition, and reduction of the Shh signal decreases hair growth (Wang et al., 2000). Nuclear factor 1 (NF1) accelerates telogen–anagen transition by inducing Shh, Wnt5a and lef1 expression, initiating anagen via activation of TGF-β2 and inhibition of p21 (Plasari et al., 2010). Noggin, c-myc and RunX1 also play roles in HF cycling (Houschyar et al., 2020).

The transition from anagen to catagen is the result of crosstalk between tumour necrosis factor α (TNF-α), vitamin D receptor (VDR) and retinoic acid (Chuma et al., 2012; Wu et al., 2014). The TNF-α signalling pathway works in conjunction with keratin 17 to promote entry to catagen by modulating apoptosis (Tong and Coulombe, 2006). Unlike TGF-β2, TGF-β1 promotes the transition from anagen to catagen. This transition can be enhanced by brain-derived neurotrophic factor (BDNF) (Foitzik et al., 2000; Peters et al., 2005). The function of VDR in HF cycling is not dependent on binding its ligands, while it acts in cooperation with β-catenin and only functions in cycling to accelerate the onset of catagen (Bikle and Christakos, 2020). In the HF of wild-type mice, FGF5 is localised in the ORS during the anagen phase. The absence of FGF5 results in prolonged anagen and the production of singularly long hair (Rishikaysh et al., 2014). Interleukin-1β, BMP2/4, neurotrophin-3 (NT-3) and NT-4 also play roles in inducing catagen (Schneider et al., 2009). The HF in anagen is considered an immune-privileged area, and attack by the autoimmune system results in CD8^+^ T cells and NKG2D^+^ cells occurring in the perifollicular area, leading to HF dystrophy and progression of catagen. JAK2-STAT3 is critical for the activation of these cytotoxic clusters (Ito et al., 2008; Xing et al., 2014).

Hair follicle cycling is a complicated process involving interactions among many signalling pathways. Although some regulatory mechanisms have been investigated, the complete process has yet to be fully elucidated.

## Characteristics of Non-Coding RNA

There are many classes of ncRNA, which constitute a large fraction of the transcriptome. By binding to target genes, RNAs and proteins, ncRNAs perform modulatory functions in most biological processes. Of the many types of ncRNA, miRNAs and lncRNAs have the most prominent regulatory roles (Yu et al., 2015).

### MicroRNA and Long Non-coding RNA Biogenesis

MicroRNAs and lncRNAs constitute the majority of ncRNAs in eukaryotes. miRNAs are typically 18–25 nucleotides long and are involved in post-transcriptional gene regulation (Ha and Kim, 2014). The biogenesis of miRNA involves several steps, starting with a precursor RNA molecule ∼1,000 nucleotides in length that is transcribed by RNA polymerase II from the genome, generating primary-miRNA (pri-miRNA). Then, the microprocessor complex containing Drosha, an RNase III, processes the pri-miRNA into precursor microRNA (pre-miRNA) inside the nucleus. A cytoplasmic RNase III, Dicer, subsequently processes the pre-miRNA, which is exported to the cytoplasm by exportin-5 as double-stranded miRNA (18–25 nucleotides). One strand of the miRNA duplex binds to its target gene along with the RNA-induced silencing complex, while the left strand of the duplex is typically degraded (Krol et al., 2010; Kim et al., 2016). The mature miRNAs can then perform their translational repression and deadenylation functions. The process outlined above is known as the canonical miRNA biogenesis pathway. Non-canonical miRNA biogenesis pathways have also been proposed, such as the mirtron pathway, in which pre-miRNAs are subjected to splicing and debranching by debranching enzyme-1, instead of being cleaved by Microprocessor (Berezikov et al., 2007; Okamura et al., 2007). In addition, the pre-miRNA Pre-miRNA-451 is too short for Dicer to cleave because of its short loop structures, and is currently the only known example of a Dicer-independent miRNA (Yang et al., 2010). Similar to other RNAs, miRNA production is controlled by RNA-binding proteins. For example, the Lin-28 protein selectively blocks maturation of the miRNA pri-let-7 by interfering with Dicer (Viswanathan et al., 2008). Furthermore, tumour protein p53 is associated with miRNA-34a in many diseases (Cortez et al., 2016; Shetty et al., 2017), and the small molecule Rubone can enhance the expression of miRNA-34a via the p53 pathway (Xiao et al., 2014).

Long ncRNAs are >200 nucleotides long and are involved in various developmental processes (Carninci et al., 2005; Lecerf et al., 2019). lncRNAs are highly similar to mRNAs in terms of their structure and maturation process, albeit that they lack an open reading frame; they are transcribed by RNA polymerase II, then capped and polyadenylated (Quinn and Chang, 2016). According to their relative position and orientation to protein-coding genes, lncRNAs can be further classified into five groups: intergenic lncRNAs, intronic lncRNAs, sense lncRNAs, antisense lncRNAs and bidirectional lncRNAs. The lncRNAs are mostly localised in the nucleus (chromatin, nucleoplasm), although some perform their functions in the cytoplasm (Shi et al., 2016; Chen et al., 2017). Moreover, many lncRNAs can be spliced into smaller functional RNA units, thus serving as vehicles for small RNAs (sRNAs) (Askarian-Amiri et al., 2011).

### Non-coding RNA Functions

The ncRNAs perform diverse regulatory functions in most biological processes; they are associated with physiology, cancer, neuropathy, immunological disorders and cardiovascular diseases (Wang et al., 2016; Adams et al., 2018; Chen X. et al., 2018; Bi et al., 2019). Thus, ncRNAs could be exploited for disease diagnosis, prognosis, therapy selection and treatment development.

MicroRNAs regulate gene expression by guiding Argonaute proteins to specific sites on mRNA 3′-untranslated regions via their ‘seed’ regions (5–8 nucleotides) (Wang et al., 2015). miRNAs modulate gene expression via two mechanisms: translational repression and mRNA degradation. The mechanism is determined by the degree of complementarity between the miRNA and its target mRNA (Yu et al., 2015); high complementarity will trigger mRNA degradation via the RNA-mediated interference pathway; otherwise, translation is repressed. Sean et al. developed a method to identify the mRNA targets of miRNAs and their binding affinities, which enabled prediction of miRNA-mediated gene repression (McGeary et al., 2019). Furthermore, small molecules can influence the function of miRNAs. For example, aminosulfonylarylisoxazole inhibits the maturation of pre-miRNA-31a into miRNA-31a (Im et al., 2017). Many other compounds that affect the functions of miRNAs to varying degrees have been discovered (Watashi et al., 2010; Pawellek et al., 2014).

Until recently, lncRNAs were dismissed as transcriptional noise. With the rapid development of high-throughput sequencing technology, the functions of many lncRNAs have been elucidated, although most remain uncharacterised. lncRNAs function by interacting with DNA, RNA or proteins. Of the numerous lncRNAs discovered, some have been well characterised. The lncRNA X-inactive specific transcript (Xist) directly interacts with the SHARP protein, which then recruits SMRT and triggers gene silencing in female mammals (McHugh et al., 2015). The lncRNA HOX transcript antisense RNA (HOTAIR) modulates miRNAs such as miRNA-127 and miRNA-206 during the metastasis of various cancers, including breast, gastric and prostate cancer (Mendell, 2016; Spokoini-Stern et al., 2020; Takei et al., 2020). Similar to miRNAs, compound 5 targets the 3′ element of the lncRNA metastasis-associated lung adenocarcinoma transcript 1 (MALAT1), providing a potential therapy for MALAT1-driven cancers (Abulwerdi et al., 2019).

### Role of Non-coding RNAs in the Regulation of Hair Follicle Development

There is accumulating evidence that the morphogenesis and regeneration of HFs described above are regulated by several types of ncRNA. Thus, the regulation of ncRNAs may determine the developmental fate of HFs. The ncRNAs with explicit functions are summarised in Tables 1, 2.

**TABLE 1:** MicroRNAs associated to HFs.

**MiRNA**	**Expression location in HFs**	**Target genes**	**Function in HFs**
miR-10a (Lv et al., 2019)	Unknown	Bmp7	DPCs proliferation ↓
miR-22 (Yuan et al., 2015; Yan et al., 2019; Cai et al., 2020)	DPCs	Lef-1, STK40, TP63, Dlx3, Foxn1, Hoxc13	HF stem cells proliferation and differentiation ↓, keratinocyte expansion and differentiation ↓
miR-24 (Amelio et al., 2013)	IRS	Tcf-3, β-catenin	Hair keratinocyte differentiation ↑
miR-26a (Ding et al., 2020)	DPCs	Smad1	DPCs proliferation ↓
miR-29a (Ge et al., 2019)	Hair matrix, IRS	Lrp6, Ctnnb1, Bmpr1a	HF stem cells proliferation ↓, matrix proliferation ↓
miR-31 (Mardaryev et al., 2010; Kim and Yoon, 2015; Luan et al., 2017)	Hair matrix, IRS, ORS	Krt16, Krt17, Dlx3, Fgf10, STK40, LATS2, Tgf-β2	Hair keratinocyte differentiation -, matrix cells proliferation ↑
miR-137 (Dong et al., 2012)	Unknown	Mitf	Hair pigmentation ↓
miR-140 (Chen et al., 2020)	Extracellular vesicles of DPCs	Bmp2	ORS proliferation↑, matrix cells proliferation ↑
miR-148a (Lv et al., 2019)	Unknown	Bmp7	DPCs proliferation ↓
miR-195 (Liu, 2015; Zhu N. et al., 2018)	DPCs	Lrp6	HF induction of DPC ↓
miR-203 (Warshauer et al., 2015; Ma et al., 2021)	Unknown	DDOST, NAE1	Not mentioned
miR-205 (Wang et al., 2013)	HF stem cells	Inpp4b, Frk, Phlda3, Inppl1	HF stem cells proliferation ↓,progenitor cell proliferation ↓
miR-214 (Ahmed et al., 2014; Du et al., 2018)	Hair matrix	EZH2, β-catenin	HF stem cells proliferation and differentiation ↓, matrix proliferation ↓, progenitor cell migration -
miR-218 (Zhao et al., 2019)	Unknown	SFRP2	HF growth ↑, hair cycle ↑
miR-339 (Li et al., 2018)	HF stem cells	Dlx5	HF stem cells differentiation ↓

*↑: promote, ↓: inhibit, -: unknown.*

**TABLE 2:** Long ncRNAs associated to HFs.

**LncRNA**	**MiRNA of axis**	**Expression location in HFs**	**Target genes**	**Function in HFs**
lncRNA-Xist (Lin et al., 2020b)	miR-424	DP *in vitro* (3D)	Shh	DPCs activity and proliferation, DP markers expression ↑
lncRN-PCAT1 (Lin et al., 2020a)	miR-329	DP *in vitro* (3D)	Wnt10b	DPCs Characteristics, DP markers expression ↑
lncRNA-5322 (Cai et al., 2019)	miR-19b	HFSC	MPK1, PI3K, AKT	HF stem cells proliferation and differentiation ↑
lncRNA-5322 (Ahmed et al., 2011; Cai et al., 2018)	miR-21	HFSC, HFs epithelium	Pten, Pdcd4, Timp3 and Tpm1, BMP	HF stem cells proliferation and differentiation ↑

*↑: promote, ↓: inhibit, -: unknown.*

Many ncRNAs modulate HF development by targeting one or multiple signalling pathways. For example, the ncRNA miRNA-214 decreases β-catenin expression directly, and can also target EZH2 to exert the same effect. Overexpression of miRNA-214 leads to abnormal HF patterns and hair formation, and this phenotype can be rescued by Wnt agonists. miRNA-214 regulates HFs by directly binding to the 3′-untranslated region of β-catenin (Ahmed et al., 2014). Similar to miRNA-214, miRNA-195-5p also modulates HF inductivity by targeting low-density lipoprotein receptor-related protein 6 (LRP6), causing inhibition of Wnt/β-catenin activation (Zhu N. et al., 2018). In an alpaca model, the expression level of the miRNA let-7b was shown to be negatively correlated with EDA expression levels in ear and back hair. Overexpression of let-7b causes reductions in EDA mRNA and protein levels, indicating that let-7b regulates HF development in alpaca cells by targeting the EDA gene, which is also associated with hypohidrotic ectodermal dysplasia (Liu et al., 2018). Let-7b also modulates EDA expression by post-transcriptionally regulating TGF-β receptor I via a different mechanism (Yan et al., 2016). Several other miRNAs are involved in HF development, including miRNA-21 (targets Pten, Pdcd4, Timp3 and Tpm1 and BMP), miRNA-24 (targets Tcf-3 and Wnt/β-catenin), miRNA-218-5p (targets SFRP2 and Wnt/β-catenin) and miRNA-125a (targets Wnt2 and Wnt/β-catenin) (Ahmed et al., 2011; Amelio et al., 2013; Chen Y. et al., 2018; Zhao et al., 2019).

The modulatory functions of lncRNAs often occur via gene methylation. Three lncRNAs, H19, RP11-766N7.3 and HOTAIR, are differentially expressed in low- and high-passage DPCs, and regulate development by triggering methylation of Wnt inhibitory factor-1, a key suppressor of the Wnt/β-catenin pathway (Liu, 2015). Recent advances in high-throughput technologies and bioinformatics software have enabled researchers to investigate ncRNAs and their potential functions in the HF. Yuan et al. identified several novel lncRNAs in Liaoning cashmere goats via high-throughput sequencing, and found that the lncRNA XLOC_008679 may affect the fineness of cashmere by targeting keratin 35 (Zheng et al., 2019). Furthermore, 13 putative lncRNAs were identified in cashmere goat secondary HFs. In the HF cycle, four lncRNAs (599528, 599518, 599511, and 599497) are upregulated during the telogen phase, while another six (599618, 599556, 599554, 599547, 599531, and 599509) are upregulated during the anagen phase, suggesting that they play relevant roles. Moreover, a network of miRNAs and their targets in the Wnt signalling pathway was constructed for each lncRNA, revealing the interplay between ncRNAs and their targets during HF development (Bai et al., 2018).

Surprisingly few studies have reported direct regulation of HF signalling pathways by lncRNAs. The lncRNA H19, which was identified in Liaoning cashmere goats via crosslinking and immunoprecipitation-sequencing, may target miRNAs (miR301a-3p, miR301b-3p, and miR766-5p) that are predicted to be involved in hair shaft formation (Zhu Y. B. et al., 2018). The H19 gene varies in terms of its methylation level throughout the HF cycle, suggesting its involvement in HF development. H19 may indirectly regulate HF cycling by acting as a vehicle for sRNAs. Protein-coding genes may be modulated by competing endogenous RNA (ceRNA) interaction networks, otherwise known as the lncRNA–miRNA–mRNA axis. For example, a transcriptomics study examined Aohan fine wool sheep at various developmental stages using Gene Ontology and Kyoto Encyclopedia of Genes and Genomes classification analysis, and showed that the lncRNA MSTRG.223165 functions in HFs via the lncRNA–miRNA-21–Sox6 axis (Zhao et al., 2020). Another study suggested that HOTAIR plays various roles via three axes: HOTAIR–miR-28-3p–IGF-1, HOTAIR–miR-126-5p–Wnt3 and HOTAIR–miR-1270–Gli family zing finger-2 (Jiao et al., 2019). Although multiple RNA interaction axes have been discovered *in silico*, the existence and functions of these axes must be confirmed experimentally; this may provide new insight into the regulatory functions of lncRNAs in the HF.

## Roles of Non-Coding RNAs in Specific Parts of the Hair Follicle

### Non-coding RNAs and Hair Follicle Stem Cells

Various types of stem cells reside in the mammalian epidermis, where they proliferate and differentiate in response to wounding to maintain skin homeostasis (Blanpain and Fuchs, 2006). HFSCs include keratinocyte progenitor cells, melanocyte progenitor cells and nestin-expressing HF-associated pluripotent (HAP) stem cells. Unlike HAPs, keratinocytes and melanocyte progenitor cells are unipotent stem cells, because they only differentiate into keratinocytes and melanocytes, respectively (Waters et al., 2007). Stem cells are located in the bulge area of the HF and are associated with HF construction and development, as well as the formation of intact HFs and hair during normal HF cycling (Amoh and Hoffman, 2017). The regulatory power of ncRNAs enables them to affect stem cell properties, further influencing HF development and regeneration.

Highly expressed miRNAs typically play critical regulatory roles in specific cell and tissue types (Andl and Botchkareva, 2015). miRNA-205 is a squamous epithelial miRNA enriched in skin progenitor and stem cells, especially in HFSCs residing in the bulge, and miRNA-205 knockout mice were reported to exhibit severe developmental defects, resulting in neonatal lethality. A lack of miRNA-205 expression leads to relatively short and mis-angled HFs, although its effect on terminal differentiation is weak. miRNA-205 ablation also hampers the proliferation of HFSCs and progenitor cells, suggesting that miRNA-205 is crucial for HF morphogenesis. Furthermore, miRNA-205 modulates the phosphoinositide 3-kinase (PI3K) pathway by directly targeting PI3K regulatory genes, indicating that the influence of miRNA-205 on HF may be mediated through the PI3K pathway (Wang et al., 2013). Another study suggested that miRNA-29a/b1 overexpression during the HF cycle shortened hairs, and eventually caused hair loss by repressing HFSC and matrix cell differentiation. The Wnt and BMP pathways are closely linked to HFSC lineage progression; miRNA-29a/b1 directly targets LRP6, ctnnb1 and Bmpr1a, thus supressing Wnt and BMP (Ge et al., 2019). *In vitro* miRNA-214 overexpression represses HFSC proliferation and differentiation, alters the cell cycle by directly targeting enhancer of zeste homolog 2 and disrupts Wnt/β-catenin signalling; however, these findings have yet to be validated *in vivo* (Du et al., 2018). Interestingly, a positive miRNA regulator of HFSCs has also been discovered; miRNA-124 was shown to promote the differentiation of HFSCs by targeting Sox9 and polypyrimidine tract binding protein 1 (Mokabber et al., 2019).

Compared to other cells in the HF, PlncRNA-1, TGF-β1, Wnt and β-catenin are all significantly downregulated in HFSCs. Transfection of HFSCs with PlncRNA-1 causes the upregulation of TGF-β1, Wnt and β-catenin, promoting HFSC proliferation and differentiation without affecting stemness. This positive effect can be blocked by the TGF-β1 inhibitor, LY2109761 (Si et al., 2018). Similar effects occur following transfection with lncRNA5322, which functions via the lncRNA5322–miRNA-21–PI3K/AKT axis. lncRNA5322 functions as a ceRNA to miRNA-19b-3p, which upregulates mitogen-activated protein kinase (MAPK) 1, thus enhancing HFSC proliferation and accelerating wound healing (Cai et al., 2018, 2019).

Exosomes of DPCs play a key role in the differentiation of HFSCs in the HF. To investigate the pathways regulated by DPC exosomes during HF cycling, Yan et al. (2019) cocultured DPCs and HFSCs. They found that miRNA-22-5p, which is highly enriched in DPC exosomes, negatively affects HFSC proliferation by directly binding to lymphoid enhancer binding factor-1 (Yan et al., 2019). miRNA-140-5p is enriched in low-passage DPC exosomes and targets BMP-2, which promotes ORS and matrix cell proliferation. This suggests a potential clinical application of exosomes (Chen et al., 2020).

### Non-coding RNAs and Dermal Papillae

Mesenchymal cells located in the DP are intimately involved in the development and regeneration of HFs. Aggregates of these cells are the precursors of the DP and release the initial signals that trigger HF development (Kollar, 1970). Previous research has suggested that transplanting lab-cultured DPs into skin could induce HF formation; however, this has failed in human subjects (Jahoda et al., 1984; Qiao et al., 2009). In a previous study, DPCs were collected from seven human individuals and used to construct a spheroid via hanging drop culture. The spheroid complexes were transplanted into mice between the foreskin dorsal epidermis and dermis, and *de novo* human HFs were observed approximately 6 weeks later (Higgins et al., 2013). This suggests that the ability of DPCs to induce HF generation is maintained in three-dimensional (3D) cultures. Therefore, DPCs play a crucial role in HF regeneration.

Based on gene chip and high-throughput sequencing data, Lv et al. (2019) constructed a dual-luciferase reporter system to investigate interactions among miRNA-148a, miRNA-10a and BMP-7 in Hu sheep cells; they found that miRNA-148a and miRNA-10a targeted and regulated BMP7 at the mRNA and protein levels. Transfection with these two miRNAs inhibited DPC proliferation via the TGF-β/Smads pathway (Lv et al., 2019).

Dermal papilla cells cultured in 3D regain their ability to induce HF formation, unlike those cultured in 2D. However, the mechanisms underlying this phenomenon are still unclear (Lin et al., 2020c). Recently, Lin et al. found that Xist, Shh and Shh-associated genes (e.g., Gli family zinc finger 1 and 2) were upregulated, while miRNA-424 was downregulated, in 3D DPC cultures. Xist functions as a ceRNA for miRNA-424, and 3D culture conditions activate the Shh signalling pathway. The HF regeneration ability of DPCs is maintained during 3D culturing due to the Xist–miRNA-424–Shh axis (Lin et al., 2020b). Moreover, the same authors also reported that lncRNA prostate cancer-associated transcript-1 (PCAT1) may also maintain the properties of DPCs and promote HF regeneration in 3D conditions via the PCAT1–miRNA-329–Wnt10a axis (Lin et al., 2020a). Another study identified a novel lncRNA (lncRNA-000133) in the secondary HFs of cashmere goats. Analysis of ceRNAs revealed that lncRNA-000133 and its related miRNAs are involved in HF development and cycling. Overexpression of lncRNA-000133 in DPCs caused the upregulation of DPC marker genes, suggesting that lncRNA-000133 contributes to the properties of DPCs (Zheng et al., 2020).

### Non-coding RNAs and Hair Follicle Pigmentation

Hair shaft pigmentation occurs via successive interactions between HF pigmentary units, which consist of follicular melanocytes, matrix keratinocytes and DP fibroblasts. Unlike epidermal pigmentation, the hair shaft pigmentation process is intermittent, occurring only during the anagen phase of the HF cycle (Slominski et al., 2005). Hair pigments are only produced within the bulb. The pigmentation process is modulated by various elements, including growth factors, the physiochemical environment of the HF and signalling pathways such as MAPK, stem cell factor/c-Kit, microphthalmia-associated transcription factor (MITF), endothelin (ET) 1, ET3/ETA and ETB (Shin and Lee, 2013; D’Mello et al., 2016; Chen T. et al., 2018).

Advances in sequencing technologies have facilitated the discovery of several miRNAs involved in pigmentation. Tian et al. (2012) used Illumina sequencing technology to investigate the sRNA profiles of white and brown alpaca skin, and discovered four differentially expressed miRNAs (miRNA-211, miRNA-424, miRNA-202, and miRNA-184); these miRNAs regulate melanogenesis by targeting genes associated with the melanogenesis pathway. In another study, the target genes of miRNA-202 in C57BL/6 black mice and BALB/c white mice were predicted. Three genes (Wnt5a, kit and tcf-7) were negatively regulated by miRNA-202, indicating a role of miRNA-202 in melanogenesis (Qu et al., 2017). Furthermore, the expression levels of miRNA-10b and miRNA-211 are significantly higher in black than white HFs in mice. Evidence suggests that miRNA-10b regulates the MAPK and Notch signalling pathways, while miRNA-211 interacts with MITF. MITF is a master regulator of melanogenesis targeted by several other miRNAs, including miRNA-25, miRNA-137, miRNA-148, miRNA-182, miRNA-218, and miRNA-340 (Bemis et al., 2008; Haflidadottir et al., 2010; Zhu et al., 2010; Yan et al., 2012; Guo et al., 2014; Zhao et al., 2017). Moreover, miRNA-137 can affect pigmentation *in vivo*; Dong et al. (2012) constructed a miRNA-137 transgenic mouse model, in which the coat colour of the mice varied according to the expression level of miRNA-137, ranging from dark black to a light colour.

Compared to miRNAs, relatively few lncRNAs have been found to be involved in melanogenesis. The lncRNA taurine upregulated gene 1 (TUG1) is downregulated following UV-B exposure, which suggests that TUG1 plays a negative role in UV-B-induced melanogenesis. TUG1 may function via the extracellular-regulated protein kinase signalling pathway, and its inhibition may upregulate melanogenesis-associated genes (Fu et al., 2019). Similar to TUG1, the lncRNA urothelial cancer associated-1 also negatively influences melanogenesis by interacting with the cAMP-response element binding protein–MITF–melanogenesis axis (Pei et al., 2020). In addition, H19 is differentially expressed between hyper-pigmented and normally pigmented skin in melasma patients. H19-knockdown melanocytes revealed that melanin synthesis is significantly increased (Kim et al., 2010).

## Clinical Translation of Non-Coding RNA-Based Treatments for Hair Follicle Regeneration

Although ncRNAs do not encode proteins, their ubiquity and robust regulatory abilities make them potential targets for clinical treatments (Curtin et al., 2018). At present, the clinical applications of ncRNAs include targets for drug treatments of cancer and other illnesses and biomarkers for tumour diagnosis and cancer prognosis (Anastasiadou et al., 2018). The complex interactive networks between ncRNAs and various regenerative signalling pathways make them attractive targets for regenerative medicine. However, obstacles such as a lack of targeted delivery methods and instability still impede the clinical translation of ncRNAs. A step that must be taken is to develop a safe and stable system to deliver ncRNAs or ncRNA modulators (ncRNA mimics or ncRNA antagonists) to their appropriate targets.

### Delivery Systems for Non-coding RNAs

The two major categories of ncRNA delivery system are viral and non-viral vectors. There are four kinds of viral vector: retroviruses, lentiviruses, adeno-associated viruses and adenoviruses. Viral vectors are efficient and can achieve relatively stable and prolonged ncRNA expression compared to non-viral vectors. Nevertheless, issues such as immunological toxicity, high mutation rates and off-target effects limit the clinical utilization of viral delivery systems (Jia and Zhou, 2005). Some biomaterials can attenuate these drawbacks, thus surmounting some of the challenges presented by viral vectors (Wang et al., 2021); however, the biomaterials are not able to fully prevent the associated hazards.

Non-viral systems include naked oligonucleotides, chemically modified oligonucleotides, lipid-based vectors, dendrimers, natural and synthetic polymers and exosomes (Miller et al., 2015). Naked oligonucleotide delivery is typically the least effective, because unmodified RNA is degraded rapidly by ubiquitous RNases. However, RNA chemical modifications can enhance the stability of antisense RNA sequences (Zhang et al., 2013). Oligonucleotide drugs with chemical modifications are currently being tested in clinical trials. For example, miravirsen (SPC3649), a locked nucleic acid antisense miRNA targeting miRNA-122, has entered human clinical trials and shows great therapeutic potential for treating Hepatitis C virus infections (Janssen et al., 2013).

Felgner et al. (1987) reported that fusogenic cationic lipids can be combined with plasmids to form liposomes, increasing the efficiency of transfection *in vitro*. Liposomes were subsequently synthesised and developed commercially, and have been widely used as transfection reagents. Until recently, however, the *in vivo* applications of liposomes have been limited due to toxicity and immunological side effects (Lv et al., 2006). To overcome these problems, neutral lipid emulsions were developed and applied to successfully treat non-small cell lung cancer (Trang et al., 2011). With the urgent need of vaccines to protect against coronavirus disease 2019 (COVID-19), some lipid-nanoparticle–mRNA-based vaccines were developed and proceeded to clinical trials (NCT04368728 and NCT04470427) (Walsh et al., 2020; Baden et al., 2021). Although their efficacy and safety were demonstrated, the possibility of reactogenicity cannot be neglected and longer-term data are required. However, these vaccines represent a major step forward for clinical application of lipids.

Dendrimers also represent attractive RNA delivery systems. In one study, a novel dendrimer complex was constructed by attaching EpDT3, a 19-nucleotide RNA aptamer, and polyethylene glycol (PEG) to the surface of a poly-amidoamine, which was used as a vector to deliver lncRNA (Tai et al., 2020). Natural and synthetic polymers are also interesting prospective delivery systems. Natural polymers include chitosan, protamine, atelocollagen and peptides. However, these polymers are more likely to elicit an immunologic response, which restricts their use. Poly(lactide-co-glycolide) (PLGA) is a synthetic polymeric material currently being considered for RNA delivery, because it can condense around oligonucleotide targets and enable controlled release, and is biodegradable and biocompatible (Danhier et al., 2012). Zhang et al. (2020) utilised monomethoxy(PEG)-PLGA-poly(L-lysine) as a miRNA delivery vector, which prolonged the circulatory time of miRNA-125a *in vivo*. As a result, PLGA is likely to be developed further as a drug delivery system.

Recently, spherical nucleic acid (SNA) has shown great promise as a drug delivery system. SNA is a nanostructure composed of chemically modified nanoparticles at its core, which is surrounded by a shell of highly arranged oligonucleotides. Unlike stranded nucleotides, SNA can enter into cells without the need for transfection reagents (Mokhtarzadeh et al., 2019). The unique structure and biological properties of SNA make it a highly promising delivery system with clinical applications (Kapadia et al., 2018). Recently, some research and trials yielded positive preliminary results in the treatment of patients with psoriasis or various skin tumours by injection of specific SNA-based drugs. The safety and efficacy of SNA have been well validated (NCT03086278) (Nemati et al., 2017; Korkmaz and Falo, 2020).

### Non-coding RNA-Based Treatments for Hair Loss

Currently, therapies for hair loss are focused on HF transplantation and drug intervention. However, the costs of transplantation are high and the effectiveness of the available drugs is disputable. On the other hand, ncRNA-based therapeutic strategies for alopecia show great clinical potential. The diagnosis and classification of alopecia are mainly based on clinical symptoms, medical history (e.g., drug use and diseases), trichoscopy and biopsies (Mubki et al., 2014a, b). Trichoscopy is a diagnostic technique widely used by dermatologists, although it requires shaving or dyeing the target area. Biopsy is still the gold standard, but is an invasive method. miRNAs and lncRNAs can be detected in body fluids and can be detected in a non-invasive manner to complete the diagnosis of alopecia. This lays a solid foundation for future ncRNA-based therapy (Panzitt et al., 2007; Sheng et al., 2019). Goodarzi et al. (2012) compared the miRNA profiles of bald and non-bold HF papillae, and found that the expression of four miRNAs (miRNA-221, miRNA-125b, miRNA-106a, and miRNA-410) was higher in the HFs of bald papillae, which suggested that these miRNAs are involved in male pattern baldness. Based on the differential expression of ncRNAs, precise interventions to slow the progression of hair loss could potentially be developed. However, further investigations are still required.

The pathogenesis of hair loss is complex and varies among individuals. For example, although autoimmune processes are known to be involved in the pathogenesis of alopecia areata, the disease aetiology is still not clear (Gilhar et al., 2012). Genome-wide miRNA analysis suggested that miRNA-30b/d was involved in alopecia areata, along with pro-inflammatory factors and natural killer cell-stimulating ligands (Ito et al., 2008; Tafazzoli et al., 2018). Similarly, in androgenetic alopecia, evidence suggests that androgen is a pathogenic driver, together with retinoid receptors (especially peroxisome proliferator-activated receptors) (Ho et al., 2019). Hair loss in androgenetic alopecia may also be attributed to abnormally expressed miRNA-133b (Deng et al., 2021). Therefore, the curative effects of non-personalised therapies are, to some extent, limited. ncRNA-based treatments can be personalised by delivering RNA mimics or antagonists tailored to the patient’s needs.

Dermal papillae are crucial centres of HF regeneration; 3D-cultured DPCs can regenerate HF (Lin et al., 2020b). miRNA-218-5p is significantly upregulated in the exosomes of 3D-cultured DPCs. Furthermore, a miRNA-218-5p mimic/polyethylenimine (PEI) or miRNA-218-5p inhibitor/PEI were injected into mouse dorsal skin. HF development was promoted in the group injected with the miRNA-218-5p mimic, but this effect was stronger in mice treated with exosomes. These observations suggested that miRNA-218-5p may be an important factor in hair growth, and the superior effect of exosomes may result from many other substances contained within them (Hu et al., 2020). In another study, human HF cells were reprogrammed into induced pluripotent stem cells by inducing miRNA-302 expression, which repressed four epigenetic factors (AOF2, AFO2, MEPC1-66 and MEPC2). This global demethylation can be reversed by adding AFO2 (Lin et al., 2011). Recently, Lee et al. (2020) constructed a complete hair-bearing skin organoid highly similar to normal human skin and entirely derived from human pluripotent stem cells. Following transplantation of the organoid HFs into nude mice, the HFs maintained their regenerative properties; to some extent, transplantation of this organoid achieved HF regeneration (Lee et al., 2020).

Non-coding RNAs have potential for treating hair loss. Research has focused more on the effects of ncRNA in one specific organ, while ncRNAs are not expressed only in specific sites (Barwari et al., 2016). Therefore, the specificity may hinder ncRNA-based therapeutics from being translated into clinical applications. Determining the most appropriate dosage, delivery system and administration route represent key points for attenuating potential adverse effects. Excessively high or low doses of ncRNA will result in off-target effects, undesired on-target effects or impairment of the curative effect (Jin et al., 2015). To eliminate these effects, the optimal dosage should be determined for clinical applications (Winkle et al., 2021). Off-target effects are major concerns in therapeutic use of ncRNAs, especially with systemic injection. Corrie et al. used the miR-29 mimic, remlarsen, to treat keloid scars through intradermal injection, and observed no obvious life-threatening toxicities (Gallant-Behm et al., 2019). Adverse reactions could be attenuated to some extent by using intradermal injection instead of systemic administration to treat skin disorders. Appropriate delivery systems are also pivotal for translating ncRNA therapeutics into clinical applications, as discussed above.

## Concluding Remarks and Future Directions

Hair loss, which is caused by many factors, is far from being cured due to its complex mechanisms. Although hair loss is not regarded as a disease in most circumstances, it significantly impacts the social life and mental health of patients and can cause depression, especially in women (Tas et al., 2018). The best strategy for dealing with hair loss would be diagnosis and treatment by regenerating HFs as early as possible. The developmental and regenerative mechanisms of the HF have been studied extensively, but the crosstalk between signalling pathways and specific modulatory mechanisms require further elucidation.

Non-coding RNAs are regulatory biomolecules that account for a large proportion of the human genome, despite not encoding proteins. Many ncRNAs are enriched in the skin and HFs, and are involved in their formation. A complex regulatory network of ncRNAs is intimately involved in HF development and regeneration. Among the various types of ncRNAs, miRNAs and lncRNAs are critical modulators of physiological and pathological processes. Recently, with the advent of novel sequencing technologies, a plethora of ncRNAs have been identified and found to regulate HF-associated mechanisms, illustrating their promise as targets for hair loss therapies. Current research exploring the regulatory mechanisms of ncRNAs in the HF tend to use high-throughput techniques (such as microarrays and RNA chips) to acquire large genetic and transcriptomics datasets, utilizing bioinformatics methods to analyse potential targets in relevant signalling pathways and verifying the ncRNA mechanisms discovered via omics analyses. However, these verification processes have largely remained at the *in vitro* phase, and *in vivo* studies are lacking. Therefore, it is necessary to characterise more ncRNAs experimentally, and to investigate the specific regulatory mechanisms of action of the discovered ncRNAs in HFs *in vivo* to develop clinically applicable ncRNA-based therapies.

During ncRNA therapy development, the drug delivery system must be considered. As discussed above, both viral and non-viral systems show promise for delivering specific RNAs; however, issues such as biotoxicity and inefficient transfection still limit their clinical progress and must be improved. Although the US Food and Drug Administration has approved several nucleic acid-based therapies for clinical use, efficacy is moderate. SNA shows promise as a robust delivery system for ncRNA-based personalised treatments. Two companies, Exicure and Allergen, are collaborating to discover and develop an SNA-based treatment for hair loss (Businesswire, 2019). The development of lipid-based vaccines for COVID-19 has also demonstrated the potential of ncRNA-based therapies. As ncRNAs are not uniquely expressed in the HF, the dosage and administration routes should also be considered carefully when employing ncRNAs to treat hair loss. Fortunately, as a skin disorder, hair loss can be treated by local administration, which can attenuate many side effects to some extent.

Non-coding RNAs, especially miRNAs and lncRNAs, are intimately involved in the regeneration and development of HF, indicating their great potential in the diagnosis and treatment of hair loss among clinical applications. In summary, although many hurdles remain, there has been a great deal of research and preclinical work regarding the potential of ncRNA-based therapies in the field of HF regeneration.

## Author Contributions

MY and XW conceived of topic for this review. All authors listed have made a substantial, direct and intellectual contribution to the work, and approved it for publication.

## Conflict of Interest

The authors declare that the research was conducted in the absence of any commercial or financial relationships that could be construed as a potential conflict of interest.

## Publisher’s Note

All claims expressed in this article are solely those of the authors and do not necessarily represent those of their affiliated organizations, or those of the publisher, the editors and the reviewers. Any product that may be evaluated in this article, or claim that may be made by its manufacturer, is not guaranteed or endorsed by the publisher.
